# Safety and efficacy of prophylactic tirofiban infusion for acute intracranial intraprocedural stent thrombosis

**DOI:** 10.1038/s41598-021-00872-9

**Published:** 2021-10-29

**Authors:** Lili Sun, Jinping Zhang, Yun Song, Wei Zhao, Meimei Zheng, Jun Zhang, Hao Yin, Wei Wang, Yao Meng, Ju Han

**Affiliations:** Department of Neurology, The First Affiliated Hospital of Shandong First Medical University and Shandong Provincial Qianfoshan Hospital, 16766 Jingshi Road, Lixia District, Jinan, 250000 People’s Republic of China

**Keywords:** Diseases, Medical research, Neurology

## Abstract

Periprocedural antithrombotic management with glycoprotein IIb/IIIa inhibitors (GPI) for intracranial artery stenting is still controversial. We sought to assess the safety and efficacy of prophylactic tirofiban infusion for acute intracranial intraprocedural stent thrombosis in routine clinical practice. From January 2013 to December 2019, consecutive patients treated with endovascular stenting for symptomatic intracranial atherosclerotic stenosis (ICAS) were identified and dichotomized by whether tirofiban was used. The efficacy and safety outcomes were compared by propensity score matching. A total of 160 consecutive patients in the tirofiban group and 177 patients in the non-tirofiban group were enrolled. Propensity score matching analysis selected 236 matched patients. One acute intraprocedural stent thrombosis (AIST) occurred in patients receiving prophylactic tirofiban, while 8 in the non-tirofiban group. The incidence of AIST in the tirofiban group was significantly lower than that in the non-tirofiban group (0.8% vs 6.8%, *P* = 0.039). The periprocedural ischemic events (8.5% vs 5.1%, *P* = 0.424), periprocedural intracranial hemorrhage (4.2% vs 0.8%, *P* = 0.219) and 30-day total mortality (3.4% vs 0%, *P* = 0.125) were not statistically different between the two groups. Compared with conventional stenting angioplasty without tirofiban, tirofiban prophylactic infusion can lower the incidence of AIST, without increasing the risk of periprocedural intracranial hemorrhage and 30-day total mortality. However, there is no superiority in reducing periprocedural ischemic events. The current study adds more important insights to the available clinical evidence on the use of tirofiban during stenting of ICAS.

## Introduction

Intracranial atherosclerotic stenosis (ICAS) is a major cause of ischemic stroke and is associated with a high risk of recurrent stroke^1–3^. The stent angioplasty could eliminate stenosis, restore blood flow, and show promise to prevent the recurrence of stroke. However, the safety and efficacy of stent-oriented angioplasty were challenged by the high incidence of periprocedural complications, of which acute intraprocedural stent thrombosis (AIST) is one of the most serious and usually poor prognostic events^4^. The reported incidence was as high as 14.6% previously during the Wingspan intracranial stent placement and much higher during stent placement in the middle cerebral artery^5,6^. In addition, the high incidence of perioperative ischemic events is also a key concern of interventional physicians. Based on the previous studies, we believe that perioperative ischemic events may be partly caused by AIST. The adequate prevention of AIST has become a topic of paramount importance commensurate with the widespread and increasing use of stent angioplasty for ICAS.

In addition to the routine use of aspirin and clopidogrel, tirofiban, a potent glycoprotein IIb/IIIa inhibitor (GPI), has been well established in acute coronary syndromes^7^. However, currently, there are very limited data pertaining to the use of tirofiban in endovascular stent angioplasty for ICAS. Therefore, the aim of this study was to assess the safety and efficacy of tirofiban compared with conventionally antiplatelet therapy only in patients who receive stent angioplasty for ICAS.

## Methods

### Study patients

We retrospectively reviewed our stroke database to identify patients who had been treated with endovascular stenting for symptomatic, severe (stenosis degree 70–99%) ICAS between January 2013 and December 2019. All the patients have recurrent strokes after aggressive medical management, so the endovascular intervention was advised. Patients with severe ICAS and dissecting aneurysm were treated at the same time, patients with intracranial tandem stenosis were treated with more than 2 stents; patients receiving treatment for intracranial multi-vessel lesions and patients with intracranial stenting due to restenosis were excluded.

All patients or their legal guardians knew the risks and benefits of endovascular treatment, including the use of tirofiban (Hengkang, Lunan Better Pharmaceutical Co., Ltd., Shandong, China), and gave informed consent before the operation. The study protocol was approved by the ethics committee of the First Affiliated Hospital of Shandong First Medical University and complied with the Declaration of Helsinki. All research was performed under the relevant guidelines and regulations. Given its retrospective nature, the study does not require registration.

### Perioperative management and intervention procedure

All patients were on dual antiplatelet agents (100 mg aspirin and 75 mg clopidogrel) daily for at least 5 days before stenting. The details of the interventional procedure have been described previously^8,9^. In brief, all endovascular procedures were performed by an experienced neurointerventionist under general anesthesia between 1 and 3 weeks after the symptoms onset of the last ischemic stroke. The stenosis degree was determined according to the Warfarin-Aspirin Symptomatic Intracranial Disease (WASID) study^3^. Device selection depended on arterial access, lesion morphology and vascular characteristics according to the operator’s experience.

The stent thrombus formed during stent implanting is defined as AIST. In our center, intraprocedural angiography was performed at about 10-min intervals, at least 40 min, for early detection of AIST. When the stent was released, the intraoperative angiography showed a shadow defect within 5 mm to the stent or within the stent, AIST was recorded. For patients diagnosed with AIST, intra-arterial bolus followed by intravenous tirofiban infusion as a rescue therapy without other methods such as additional angioplasty or stenting was adopted as described in our previous study^8^ and remedial recanalization was evaluated by modified Thrombolysis In Cerebral Infarction (mTICI) scale and Arterial Occlusive Lesion (AOL) scale^10^.

The proactively use of tirofiban or not was at the interventionists’ discretion according to the lesions’ characteristics (such as extremely eccentric lesions, a lesion of > 15 mm in length, degree of stenosis > 90%, and features based on High-resolution magnetic resonance imaging (HRMRI) including diffuse distribution, intraplaque haemorrhage and strong enhancement), the risk of periprocedural ischemic complications and all factors associated with risk of intracranial hemorrhage (ICH). If used, the application protocol was as follows: Tirofiban was administered intravenously at a dose of 0.1 µg/kg/min immediately after puncture of femoral artery, and continued for 30 min postoperatively, except for intraoperative or postoperative hemorrhagic complications.

All patients underwent non-enhanced computed tomographic (CT) scans immediately after the procedure after stenting. Appropriate imaging techniques (CT or magnetic resonance imaging (MRI)) were used to confirm the presence of ischemic or hemorrhagic complications if patients had any signs of neurological deterioration at any time within 30 days after the intervention.

### Neurological classifications and neurological evaluations

Periprocedural neurological complications (complications within 7 days after intervention) include periprocedural ischemic events and ICH. The ischemic events were classified as transient ischemic attacks (TIA) and cerebral infarction (minor and major stroke). TIA, minor stroke and major stroke were determined according to the measurements of acute cerebral infarction proposed by Brott et al.^11^. In addition, cerebral infarction was further classified according to the etiology into perforating artery occlusive cerebral infarction, cerebral embolism, hypoperfusion type or mixed type. Recurrent ischemic events were defined as any focal neurological symptoms related to the corresponding vascular territory and unrelated to ICH based on CT imaging.

ICH was categorized according to the Heidelberg Bleeding Classification (HBC), which was proposed at a consensus meeting of leading stroke researchers to provide a generally accepted and more differentiated definition of hemorrhage following endovascular therapy^12,13^. ICH was furthermore classified as symptomatic intracerebral hemorrhage (sICH) or asymptomatic intracranial hemorrhage, in compliance with operational guidelines of the HBC. Recurrent ICH was defined as all-cause of recurrent hemorrhage confirmed by CT or MRI.

A complete neurological examination was performed by independent neurologic team members using the NIHSS score before the procedure, immediately and one week after the procedure according to our hospital stroke protocol. Recurrent ischemic and hemorrhagic events that occurred within 30 days were obtained in the outpatient clinic or by telephone by trained and masked research neurologists who were blinded to patients characteristics and treatment assignment. If necessary, brain imaging tests including MRI or CT were obtained in patients with new symptoms.

### Data collection

Data were collected from the patient medical records on baseline demographics, vascular risk factors, location of the lesion, NIHSS score at admission and 7 days after the procedure, procedural details, periprocedural complications, recurrent ischemic and hemorrhage events within 30 days. Deaths due to any cause within 30 days were recorded as well.

### Evaluation of efficacy and safety outcomes

The primary efficacy outcomes were the incidence of AIST, periprocedural ischemic events and recurrent ischemic events within 30 days. The secondary efficacy outcome was the NIHSS score 7 days after the procedure. The safety outcomes included the occurrence of periprocedural ICH, periprocedural systemic bleeding (severe extracerebral bleeding requiring additional therapy such as transfusion or surgical intervention), recurrent ICH and total mortality during 30-day follow-up.

### Statistical analysis

Statistical analyses were conducted using SPSS version 24.0 for Windows (IBM, Armonk, New York). Continuous variables were presented as the mean ± standard deviation (SD) or as the median (interquartile range) and compared using Student *t* test or Mann–Whitney *U* test. Categorical variables were presented as numbers (percentages) and compared by χ2 or Fisher exact test. Propensity score matching of patients with and without of tirofiban was performed using a 1:1 matching algorithm with a caliper distance of 0.02 with baseline data, stenosis location, stenosis degree, stent length and diameter, and residual stenosis after intervention as covariates. The matched cohorts were then compared using McNemar’s test for categorical variables and the Wilcoxon signed-rank test for numerical variables to test the differences in baseline characteristics and outcomes.

### Ethics declarations

The study protocol was approved by the ethics committee of the First Affiliated Hospital of Shandong First Medical University and complied with the Declaration of Helsinki. Written informed consent was obtained from the patients or their legal guardians. Given its retrospective nature, the study does not require registration.

## Results

### General subject characteristics

**Figure 1:**
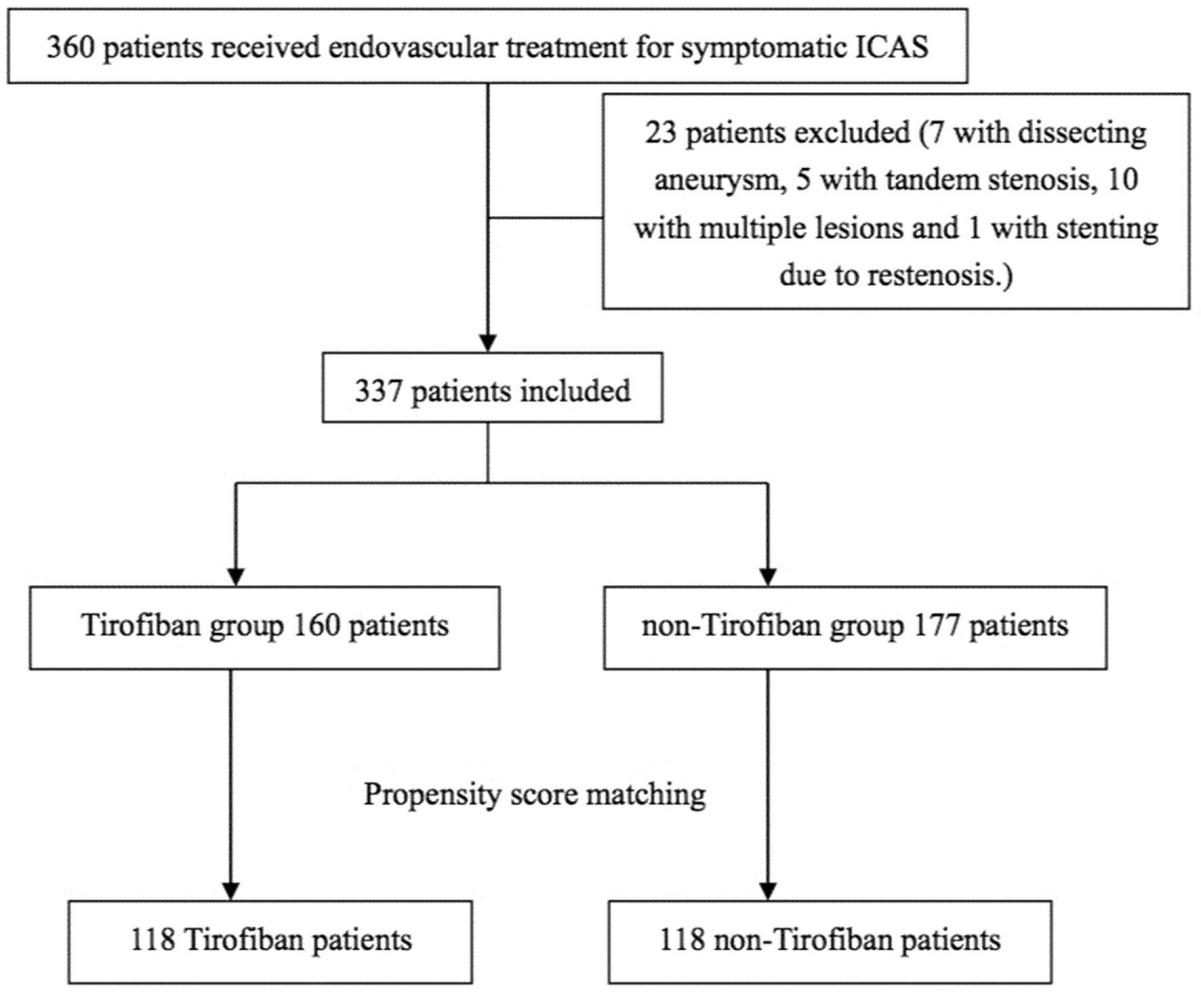
Patient flowchart. Flowchart of the study. *ICAS* Intracranial atherosclerotic stenosis.

A total of 360 patients treated with endovascular intervention for symptomatic severe ICAS were enrolled between January 2013 and December 2019. Twenty-three patients were excluded. At last, 337 patients were finally included, with 160 in the Tirofiban group and 177 in the non-Tirofiban group. Of the 337 patients, none had postoperative stenosis more than 40%. The postoperative stenosis > 10% was found in 10 of these patients. After PSM, 236 patients were matched, with 118 patients in each group. The patient flowchart is illustrated in Fig. 1.

### Baseline, periprocedural and outcome characteristics of the patients

Before PSM, there were no significant differences in baseline characteristics between the two groups, except for a significantly higher rate of hypertension and male in the non-Tirofiban group, and some parameters such as stenosis location, stent length and stenosis degree before the intervention that might influence the intervention outcome (Table 1).

**Table 1:** Baseline, periprocedural, and outcome characteristics of the patients before PSM.

	All (n = 337)	Tirofiban group (n = 160)	Non-tirofiban group (n = 177)	*P* value
**Demographics**				
Age, yr (range)	60.3 ± 8.7 (27, 82)	59.4 ± 8.9 (27, 80)	61.0 ± 8.4 (40, 82)	0.092
Male	249 (73.9)	110 (68.8)	139 (78.5)	0.041
**Medical history**				
Hypertension	242 (71.8)	105 (65.6)	137 (77.4)	0.016
Diabetes mellitus	141 (41.8)	59 (36.9)	82 (46.3)	0.079
Atrial fibrillation	4 (1.2)	2 (1.3)	2 (1.1)	1.000
Coronary artery disease	26 (7.7)	11 (6.9)	15 (8.5)	0.583
Smoking	139 (41.2)	64 (40)	75 (42.4)	0.659
Family history	50 (14.8)	27 (16.9)	23 (13)	0.317
**Stenosis location**				0.012
Anterior circulation	161 (47.8)	88 (55)	73 (41.2)	–
Posterior circulation	176 (52.2)	72 (45)	104 (58.8)	–
Admission NIHSS	1 (0, 3)	1 ((0,3)	1(0,4)	0.669
**Interventional parameter**				
Stenosis degree before intervention, %	85 (80,90)	90 (80,90)	80 (80,90)	0.000
Stent length, mm	15 (13,15)	15 (15,15)	13 (9,15)	0.000
Stent diameter, mm	3 (3,3.5)	3 (3,3.5)	3 (3,3.5)	0.139
Stenosis degree after intervention, %	0 (0,0)	0 (0,0)	0 (0,0)	0.286

PSM indicates Propensity score matching. Family history indicates history of cardiovascular and cerebrovascular diseases. TICI indicates Thrombolysis in Cerebral Infarction. Values are mean ± SD or median (interquartile range), or n (%).

### Propensity score matching

**Figure 2:**
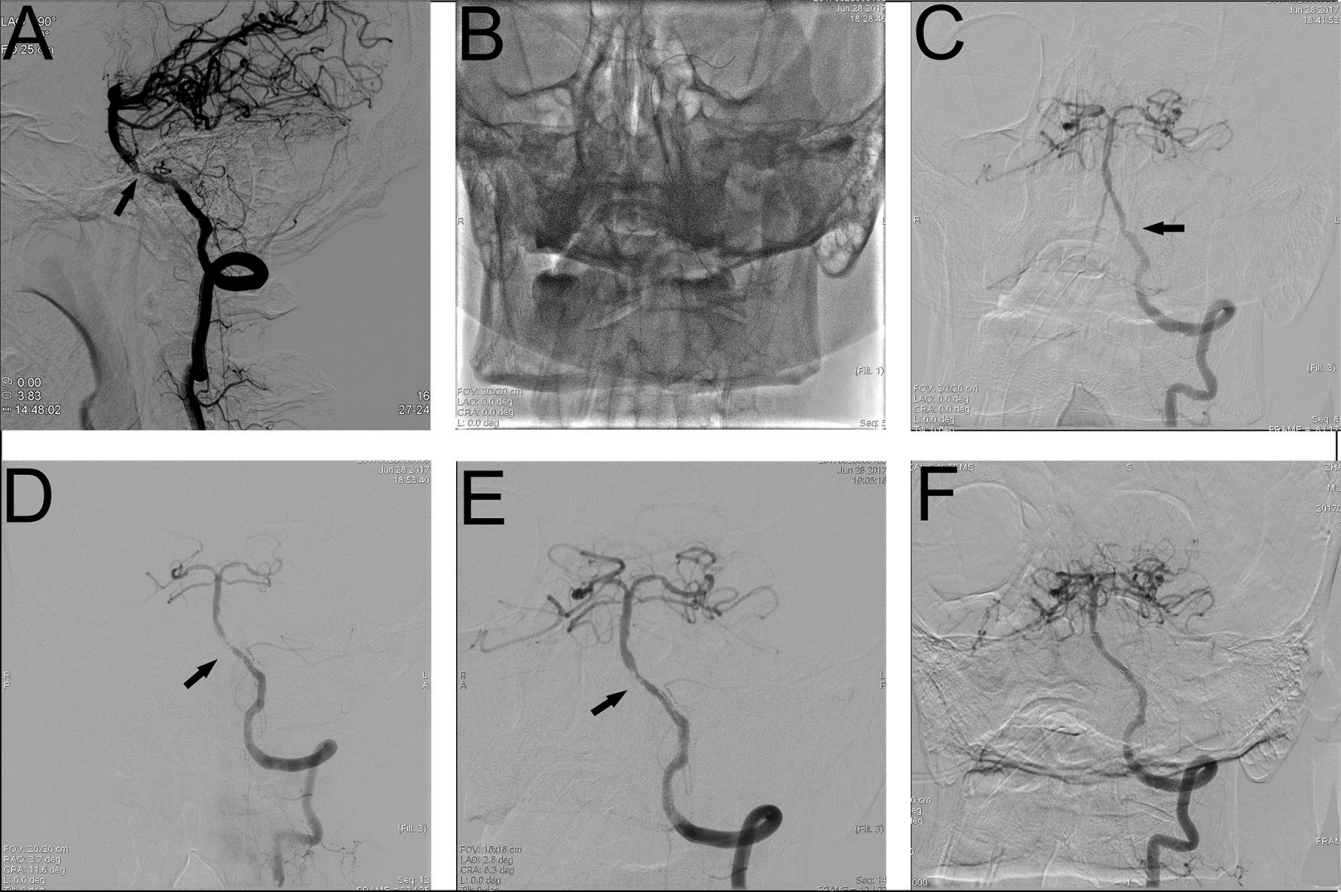
Intraoperative cerebral angiography results of AIST patients. (**A**) Preoperative angiogram showing severe basilar artery stenosis (arrow). (**B**) Conventional balloon predilation. (**C**) Angiography immediately after stent implantation showed normal blood flow in vertebrobasilar artery with stent implantation. (**D**, **E**) Delayed angiography revealed a stent thrombosis with limited blood flow. Stent tines are visible superior to the stagnant contrast. (**F**) Complete recanalization after treatment with tirofiban.

After PSM, the baseline and interventional parameters were well balanced between the two groups (Table 2). Overall, there were 9 AIST occurred among the 236 patients, 1 in the Tirofiban group and 8 in the non-Tirofiban group. All patients with AIST were successfully rescue treated by tirofiban (mTICI3, AOL3) (Fig. 2). There were 16 periprocedural ischemic events, 10 minor strokes, 6 major strokes. According to the etiology, 9 patients had perforating artery occlusion, 3 had embolization, and the infarction lesions enlarged in 4 patients due to hemodynamic disturbance. In the Tirofiban group, there were 7 minor strokes, 3 major strokes (1 neurologically-related death from a failed attempt to lyse an occluded vertebral artery perforation and the patient die 12 days after the procedure). In the non-Tirofiban group, there were 3 minor strokes, 3 major strokes. There were no recurrent ischemic events in the territory of the stented artery within 30 days.

**Table 2:** Baseline and periprocedural characteristics of the matched patients.

	All (n = 236)	Tirofiban group (n = 118)	Non-tirofiban group (n = 118)	*P* value
**Demographics**				
Age, yr (range)	60.7 ± 8.2 (40, 80)	60.9 ± 8.6 (40, 80)	60.6 ± 7.7 (41, 78)	0.771
Male	166 (70.3)	83 (70.3)	83 (70.3)	1.000
**Medical history**				
Hypertension	170 (72.0)	87 (73.7)	83 (70.3)	0.665
Diabetes mellitus	98 (41.5)	50 (42.4)	48 (40.7)	0.896
Atrial fibrillation	3 (1.3)	1 (0.8)	2 (1.7)	1.000
Coronary artery disease	19 (8.1)	11 (9.3)	8 (6.8)	0.583
Smoking	92 (39.0)	46 (39.0)	46 (39.0)	1.000
Family history	35 (14.8)	19 (16.1)	16 (13.6)	0.728
**Stenosis location**				1.000
Anterior circulation	120 (50.8)	60 (50.8)	60 (50.8)	–
Posterior circulation	116 (49.2)	58 (49.2)	58 (49.2)	–
Admission NIHSS	1 (0,3)	1 (0,3)	1 (0,3.25)	0.788
**Interventional parameter**				
Stenosis degree before intervention, %	90 (80,90)	90 (80,90)	90 (80,90)	0.755
Stent length, mm	15 (15,16.5)	15 (15,16.5)	15 (13,20)	0.090
Stent diameter, mm	3 (3,3.5)	3 (3,3.5)	3 (2.5,3.5)	0.219
Stenosis degree after intervention, %	0 (0,0)	0 (0,0)	0 (0,0)	0.918

Values are mean ± SD or median (interquartile range), or n (%).

Periprocedural ICH occurred in 5 (4 parenchymal hematoma, one subdural hemorrhage. 3 of these 5 cases were sICH.) patients in the Tirofiban group and 1 (asymptomatic SAH) patient in the non-Tirofiban group. According to clinical and imaging characteristics, 4 cases in the tirofiban group were considered to be caused by hyperperfusion, 1 case was of unknown etiology. The case of ICH in the non-Tirofiban group was caused by vessel rupture during stenting. All periprocedural ICH were detected on CT on the first day after the procedure except for one patient who suffered fatal ICH 3 days after intervention (The patient died 9 days later.). Two patients with ICH underwent surgical borehole drainage. No other ICH occurred within 30 days. In addition to the above deaths, two other patients died in Tirofiban group, one of whom died of arrhythmia on the second day after intervention. The other one died of pulmonary interstitial fibrosis and pneumonia 26 days after the intervention. No systemic bleeding requiring transfusion or surgery occurred in the 2 groups.

Therefore, the incidence of AIST in the Tirofiban group was significantly lower than that in the non-Tirofiban group (0.8% [1/118] vs 6.8% [8/118], *P* = 0.039). However, although the absolute incidence of the 30-day periprocedural ischemic events (8.5% [10/118] vs 5.1% [6/118], *P* = 0.424) and periprocedural ICH (4.2% [5/118] vs 0.8% [1/118], *P* = 0.219) were higher in the Tirofiban group than those in the non-Tirofiban group, there were no statistical differences. In addition, for 30-day total mortality, there was no statistically significant difference between the two groups (3.4% [4/118] vs 0% [0/118], *P* = 0.125). Also, there was no statistically significant difference in NIHSS score 7 days after the procedure between the two groups (*P* = 0.069) (Tables 2, 3).

**Table 3:** Efficacy and safety end points of the matched patients.

	All (n = 236)	Tirofiban group (n = 118)	Non-tirofiban group (n = 118)	*P* value
**Periprocedural complications**				
AIST	9 (3.8)	1 (0.8)	8 (6.8)	0.039
Periprocedural ischemic events	16 (6.8)	10 (8.5)	6 (5.1)	0.424
TIA	0	0	0	–
Ischemic stroke	16 (6.8)	10 (8.5)	6 (5.1)	0.424
Periprocedural ICH	6 (2.5)	5 (4.2)	1 (0.8)	0.219
Systemic bleeding	0	0	0	–
Post-procedural NIHSS (7d )	1 (0,3)	1 (0,3)	1 (0,2)	0.069
**Follow-up results (30 d)**				
Recurrent ischemic events	0	0	0	–
Recurrent ICH	0	0	0	–
Total mortality	4 (1.7)	4 (3.4)	0 (0)	0.125

Values are mean ± SD or median (interquartile range), or n (%).

## Discussion

The aim of angioplasty and stenting is to restore optimal blood flow in narrowed arteries, in order to prevent further ischemic events. However, despite the routine use of antiplatelet therapy and heparinization in conjunction with stent angioplasty, AIST and thromboembolism are still ineluctable, which has impeded the efficacy of stenting. The incidence of AIST during coronary interventional therapy was 0.5–1.2%^14,15^, and the incidence of AIST during intracranial stenting was much more higher^5,6^. In the post hoc analysis of the SAMMPRIS trial, of the 21 ischemic events, 1 was mixed embolic and perforator territory owing to AIST, 1 had a probable stent thrombosis at 6 days^16^. Therefore, the prevention of AIST is important to decrease neurologic complications.

In our center, all patients underwent diagnostic angiogram to confirm stenosis, and their angiographic characteristics were evaluated before the endovascular stenting procedure. Prophylactic tirofiban infusion was recommended based on the lesions’ characteristics, such as extremely eccentric lesions, a lesion of > 15 mm in length, degree of stenosis > 90%, and features shown on HRMRI including diffuse distribution, intraplaque haemorrhage and strong enhancement.

Numerous coronary interventional trials have evaluated the use of GPI and demonstrated a significant reduction in ischemic complications associated with angioplasty and stent placement^17,18^. In addition, GPI were frequently used for carotid revascularization in the era preceding protection devices introduction, but they are nowadays mainly reserved for acute neurovascular procedures^19–22^. Recently, several studies have reported on the safety and efficacy of tirofiban in combination with emergency endovascular angioplasty and stenting in patients with acute ischemic stroke. Baek et al.^23^ reported that the use of intravenous tirofiban for 12 h was associated with decreased risk of early reocclusion of treated arteries, with no increased risk of hemorrhage after emergent angioplasty, with or without stenting. Lee et al.^24^ reported that acute stenting with subsequent GPI was not associated with an increased risk of ICH or in-hospital death.

For elective intracranial vasculature endovascular therapy, most studies of tirofiban have focused on the prophylactic or rescue treatment of intraprocedural thromboembolic events during coil embolization of intracranial aneurysms and it is increasingly recognized that the use of GPI may be safe and effective^25,26^.

However, available data assess tirofiban as a prophylactic agent with conventional antiplatelet therapy for angioplasty of intracranial artery are lacking. Building upon these data and evidences supporting the safety and usefulness of tirofiban, we have gradually used tirofiban during stent angioplasty in selected high-risk ICAS cases.

In this proof-of-concept study, we explored the safety and efficacy of proactively intravenous administration of tirofiban in the subset of patients who received stent angioplasty for ICAS. Our single-center experience demonstrated that as compared to the baseline demographics, vascular risk factors, interventional parameter matched local cohort, prophylactic tirofiban infusion appears to reduce the risk of AIST.

The benefit of controlling AIST by local or systemic medication must be balanced against the increased risk of ICH. A prerequisite for such a treatment option is the easy application and short half-life of the drug. The GPI tirofiban seems to fulfill these requirements, at least in part^27^. Tirofiban binds specifically and reversibly to the platelet GPIIb/IIIa receptor with a short half-life in plasma (~ 1.6 h) and the prolonged bleeding time can be normalized within 4 h after discontinuation^28–30^. This differentiates tirofiban from other GPI, such as abciximab, which irreversibly binds to the GPIIb/IIIa receptor with a much longer half-time (8 + h). As a matter of fact, AbESTT was stopped prematurely due to higher rates of bleeding complications^31^. Whereas promising data from the SaTIS trial showed that tirofiban in acute stroke patients was safe^32^. Even so, the principal and most serious adverse effect of drugs that inhibit GPIIb/IIIa receptors is bleeding.

In our study, we observed 6 periprocedural ICH, 5 in the tirofiban group. Although the absolute incidence of periprocedural ICH in the Tirofiban group was higher than that in the non-Tirofiban group. However, there was no statistical difference. Furthermore, there was no statistical difference between the two groups in the 30-day total mortality. Even so, concerning the safety outcome, there is a need for a controlled, prospective trial to clarify this safety aspect.

In addition, in this retrospective study, all ischemic events occurred within 7 days after the procedure and the mechanism of infarction in most patients were perforating artery occlusion. Perforator stroke attributable to the compromise of perforating arteries remains a challenge in intracranial atherosclerotic stenosis stenting angioplasty. Perforator stroke occurs as a result of the snow plow effect, which cannot be prevented by tirofiban. This may be the reason why there is no significant difference in periprocedural ischemic stroke between the two groups.

To our best knowledge, our study is the first to report specifically on the use of prophylactic tirofiban in the setting of stent angioplasty for ICAS and the largest experience with this agent in the intervention of ischemic cerebrovascular disease thus far. Tirofiban infusion might be considered a viable alternative prophylactic for AIST during stent implantation in patients with ICAS. According to the current findings, we boldly deem that this new strategy, prophylactic tirofiban infusion in stent angioplasty for ICAS may be safe and effective, especially in terms of reducing AIST. This study provides a standardized protocol of prophylactic tirofiban infusion in stent angioplasty for ICAS and demonstrated the incidence of AIST was effectively lower, making this protocol widely generalizable. However, this hypothesis still needs to be corroborated by large, prospective, randomized trials.

## Limitations

Our study has some limitations. First, this is a retrospective, nonrandomized cohort study in one academic stroke center. Despite applying propensity score matching analysis to balance the potential covariates, it is unlikely to be well-matched with randomized controlled trials. Second, all enrolled patients are Chinese, and therefore the results may not apply to other ethnicities. Third, the choice of treatment method was based on the experience of the operator, which might lead to selection bias. Fourth, we did not conduct a subgroup analysis focus on stenosis location of anterior and posterior circulation due to the small matched sample size. Fifth, the follow-up time was short, so the results should be extrapolated with caution to long-term follow-up. However, one of the goals of this study is to gather preliminary data for future studies.

## Conclusions

Compared with conventional stenting angioplasty without tirofiban, tirofiban prophylactic infusion can lower the incidence of AIST, without increasing the risk of periprocedural ICH and 30-day total mortality. However, there is no superiority in reducing periprocedural ischemic events. The current study adds more important insights to the available clinical evidence on the use of tirofiban during stenting of ICAS. These findings should be interpreted cautiously because of the aforementioned limitations, and further prospective randomized studies are warranted to confirm these findings.

## Data Availability

Any additional information regarding our neurointerventional database will be provided after an appropriate request.

## References

[1] Wang Y (2014). Prevalence and outcomes of symptomatic intracranial large artery stenoses and occlusions in China: The Chinese Intracranial Atherosclerosis (CICAS) Study. Stroke.

[2] Jung JM (2012). Predictors of recurrent stroke in patients with symptomatic intracranial arterial stenosis. Stroke.

[3] Chimowitz MI (2005). Comparison of warfarin and aspirin for symptomatic intracranial arterial stenosis. N. Engl. J. Med..

[4] Alurkar A, Karanam LSP, Oak S, Nayak S, Sorte S (2013). Role of balloon-expandable stents in intracranial atherosclerotic disease in a series of 182 patients. Stroke.

[5] Lawson MF (2010). Acute intraprocedural thrombus formation during wingspan intracranial stent placement for intracranial atherosclerotic disease. Neurosurgery.

[6] Lee TH (2005). Preliminary results of endovascular stent-assisted angioplasty for symptomatic middle cerebral artery stenosis. AJNR Am. J. Neuroradiol..

[7] Van’t Hof A, Valgimigli M (2009). Defining the role of platelet glycoprotein receptor inhibitors in STEMI: Focus on tirofiban. Drugs.

[8] Sun L (2020). Safety and Efficacy of tirofiban in rescue treatment for acute intracranial intraprocedural stent thrombosis. Front Neurol..

[9] Han J (2019). Drug-coated balloons for the treatment of symptomatic intracranial atherosclerosis: initial experience and follow-up outcome. J. Neurointerv. Surg..

[10] Zaidat OO (2013). Recommendations on angiographic revascularization grading standards for acute ischemic stroke: A consensus statement. Stroke.

[11] Brott T (1989). Measurements of acute cerebral infarction: A clinical examination scale. Stroke.

[12] Neuberger U (2017). Classification of bleeding events: Comparison of ECASS III (European Cooperative Acute Stroke Study) and the new Heidelberg Bleeding Classification. Stroke.

[13] von Kummer R (2015). The Heidelberg Bleeding Classification: classification of bleeding events after ischemic stroke and reperfusion therapy. Stroke.

[14] Biondi-Zoccai GG (2005). Validation of predictors of intraprocedural stent thrombosis in the drug-eluting stent era. Am. J. Cardiol..

[15] Xu Y, Qu X, Fang W, Chen H (2013). Prevalence, correlation and clinical outcome of intra-procedural stent thrombosis in patients undergoing primary percutaneous coronary intervention for acute coronary syndrome. J. Interv. Cardiol..

[16] Derdeyn CP (2013). Mechanisms of stroke after intracranial angioplasty and stenting in the SAMMPRIS trial. Neurosurgery.

[17] EPILOG Investigators. Platelet glycoprotein IIb/IIIa receptor blockade and low-dose heparin during percutaneous coronary revascularization. *N. Engl. J. Med*. **336,** 1689–1696 (1997). 10.1056/NEJM199706123362401 9182212

[18] ESPRIT Investigators. Enhanced Suppression of the Platelet IIb/IIIa Receptor with Integrilin Therapy. Novel dosing regimen of eptifibatide in planned coronary stent implantation (ESPRIT): A randomised, placebo-controlled trial. *Lancet* **356,** 2037–2044 (2000). 10.1016/S0140-6736(00)03400-0 11145489

[19] Zhao W (2017). Low-dose tirofiban improves functional outcome in acute ischemic stroke patients treated with endovascular thrombectomy. Stroke.

[20] Goh DH, Jin SC, Jeong HW, Ha SY (2016). Mechanical solitaire thrombectomy with low-dose booster tirofiban injection. Neurointervention.

[21] Kapadia SR (2001). Initial experience of platelet glycoprotein IIb/IIIa inhibition with abciximab during carotid stenting: A safe and effective adjunctive therapy. Stroke.

[22] Schneiderman J (2000). Abciximab in carotid stenting for postsurgical carotid restenosis: Intermediate results. J. Endovasc. Ther..

[23] Baek BH (2021). Intravenous tirofiban infusion after angioplasty and stenting in intracranial atherosclerotic stenosis-related stroke. Stroke.

[24] Lee JI (2017). Safety of bridging antiplatelet therapy with the gpIIb-IIIa inhibitor tirofiban after emergency stenting in stroke. PLoS ONE.

[25] Liang XD (2016). Safety and efficacy of a new prophylactic tirofiban protocol without oral intraoperative antiplatelet therapy for endovascular treatment of ruptured intracranial aneurysms. J. Neurointerv. Surg..

[26] Kang HS (2008). Intra-arterial tirofiban infusion for thromboembolism during endovascular treatment of intracranial aneurysms. Neurosurgery.

[27] Bizzarri F (2001). Perioperative use of tirofiban hydrochloride (Aggrastat) does not increase surgical bleeding after emergency or urgent coronary artery bypass grafting. J. Thorac. Cardiovasc. Surg..

[28] Jauch EC (2013). Guidelines for the early management of patients with acute ischemic stroke: A guideline for healthcare professionals from the American Heart Association/American Stroke Association. Stroke.

[29] Ferguson JJ, Waly HM, Wilson JM (1998). Fundamentals of coagulation and glycoprotein IIb/IIIa receptor inhibition. Am. Heart J..

[30] Seitz RJ (2003). Thrombolysis with recombinant tissue plasminogen activator and tirofiban in stroke:preliminary observations. Stroke.

[31] Adams HJ (2008). Emergency administration of abciximab for treatment of patients with acute ischemic stroke: results of an international phase III trial: Abciximab in Emergency Treatment of Stroke Trial (AbESTT-II). Stroke.

[32] Siebler M (2011). Safety of tirofiban in acute ischemic stroke: the SaTIS Trial. Stroke.

